# Depressive symptomatology in Brazil: perspectives of statistical and psychometrics analyses of the PHQ-9 at four time-points (2020–2023) in the COVID-19 pandemic

**DOI:** 10.3389/fpsyg.2025.1440054

**Published:** 2025-02-05

**Authors:** Andre Faro, Daiane Nunes, Derek Falk

**Affiliations:** ^1^Department of Psychology, GEPPS/UFS, Federal University of Sergipe, São Cristóvão, Brazil; ^2^Case Comprehensive Cancer Center, Case Western Reserve University School of Medicine, Cleveland, OH, United States

**Keywords:** depression, PHQ-9, psychometric properties, normative score assessment, social distribution, COVID-19

## Abstract

The present research assessed the psychometric properties of the Patient Health Questionnaire-9 (PHQ-9) through an examination of its internal structure, invariance analysis, and standardization. Social distribution analyses of the measure were conducted using linear and binomial logistic regression. The sample consisted of 10,069 adults from all 27 states in Brazil. The data were obtained through four collections across different years of the COVID-19 pandemic (2020, 2021, 2022, and 2023), using independent samples. Confirmatory Factor Analysis (CFA) indicated that the measure is unidimensional with satisfactory fit indices. The model was invariant in relation to the variables investigated at four different levels (configural, metric, scalar, and strict). The standardization supported hypothetical cut scores indicating the severity of depressive symptoms, categorized as very low (0 to 6), low (7 to 13), moderate (14 to 19), high (20 to 23), and very high (≥ 24). We found that sex/gender, skin color/ethnicity, age, education level, and year of the pandemic were predictors of depressive symptoms in the adjusted linear regression analysis. The logistic regression showed variables with higher chances for a positive screening diagnosis of depression, with adjusted Odds Ratio as follows: years 2021 (OR_adj_ = 1.275) and 2023 (OR_adj_ = 1.409), women (OR_adj_ = 1.900), *Pardos* individuals (OR_adj_ = 1.252), education up to high school (OR_adj_ = 1.272), being a northeast region resident (OR_adj_ = 2.127), and younger people (OR_adj_ = 1.716). The findings of this study indicate the suitability of the PHQ-9 for assessing depression in the population and recommend its use for monitoring depressive symptoms in the coming years in Brazil. Clinical implications include developing interventions to address the psychological impact of this and any future health crises.

## Introduction

Every decade, the world prepares for a hypothetical pandemic-like health crisis—a phenomenon closely monitored by the World Health Organization (WHO). The European Commission’s Chief Scientific Advisory Group (ECCCSAG) is a dedicated committee that actively monitors and manages these proposals. Beginning in 2020, the global community experienced unique stressors due to the COVID-19 pandemic, one of the most devastating pandemics in recent history (Hiscott et al., 2020; Piret and Boivin, 2021; Smallwood, 2023). COVID-19 could be on its way to becoming an historic event, joining the larger pandemics of past centuries such as the Spanish flu, which have been extensively studied for their far-reaching effects (Demertzis and Eyerman, 2020; Moura et al., 2022; Nii-Trebi et al., 2023).

Given the magnitude of the COVID-19 outbreak, various delayed effects of the adaptive strain that people have endured over the battles of previous years are expected primarily for survival (Ashby et al., 2022; Kira et al., 2021). A significant increase in fatigue, loneliness (Mansueto et al., 2021) covid-anxiety syndrome (Alhakami et al., 2023; Mansueto et al., 2022) distress (Brailovskaia et al., 2021) as well as a decrease on well-being (Mansueto et al., 2024) have been observed across different samples and across different countries during COVID-19 pandemic. Due to the accumulation of stressors and organic as well as mental strains at various levels, vulnerabilities to certain diseases or disorders emerge (Haight et al., 2023). Previous findings in disaster Psychology provide evidence of the challenging times that follow in attempting to return to the new normal or even establishing new parameters for psychological adjustment. These adjustments involve incorporating new difficulties as part of daily life from then onwards (Nielsen et al., 2019). While we are moving towards declaring the end of the pandemic, there are still anticipated effects related to the pandemic especially concerning psychological adjustment and the mental health of the population (Gradidge et al., 2023; Kazlauskas and Quero, 2020). Health Psychology has fundamental contributions to understanding what impacted the population during this period, under what conditions, how it influenced their behaviors towards the pandemic, and how they stand today in terms of mental health and psychological adjustment. This focus frames the primary aims of this study.

Health Psychology contributes to studies aimed at assisting in planning actions that structure necessary care using an epidemiological perspective (Taylor, 2018). Actions such as addressing vaccine refusal are already being tackled by health belief models (Limbu et al., 2022; Suess et al., 2022) as well as modifying behaviors (Smail et al., 2021). Recommendations or protocols for identifying and managing depressive symptoms are also incorporated as they are a clinical condition often linked to adjustment difficulties during the pandemic (Lakhan et al., 2020). All these actions demonstrate potential applications to deal with post-COVID-19 crisis aspects as well as future demands (Esterwood and Saeed, 2020) requiring a current understanding of what transpired over the past years. Monitoring aspects of the population’s mental health throughout the pandemic contributes to understanding acute or chronic effects, potentially cumulative, that exacerbate the burden of psychological adjustment (Breslau et al., 2021). Research with this objective will help clarify the primary long-term impact on mental health across different individuals and groups.

Depressive symptoms are among the most studied mental health outcomes of the pandemic (Renaud-Charest et al., 2021). In various countries, depression has been monitored since 2020 (Dettmann et al., 2022; Mahmud et al., 2023; McPherson et al., 2021; Salari et al., 2020) given its association with greater strain and behavioral efforts to cope with the constant shocks that emerged daily with the pandemic’s grim news. The country’s major TV networks provided 24/7 coverage, seven days a week of all COVID-19 movements and statistics. Undoubtedly, it was the largest social network (or coverage) surrounding a disaster in human history. Never had there been such immediate, widespread transmission of so much misinformation in such a short time period.

The public health measures adopted to mitigate the spread of the coronavirus have had significant impacts on mental health protective structures, such as social support, routine, and access to resources like food, leisure, and performance (Shader, 2020). These changes in social dynamics are expected to induce alterations in people’s behavior, including modifications in sleep patterns, eating habits, substance use, and social interaction (Wu et al., 2021); aspects directly linked to depressive symptoms (Turna et al., 2021). Other factors contributed to an increase in depression in the population, such as high unemployment rates (Lee et al., 2021), loss of loved ones (Reitsma et al., 2021), financial insecurity (Wilson et al., 2020), social isolation (Gorenko et al., 2021), among others. Research in various countries reported high rates of depression during the COVID-19 outbreak. A study conducted in China with 14,493 individuals found that one in 20 adults had a suggestive depression score with a prevalence of 6.3% (Liu et al., 2022). The authors also noted that urban residents and nurses were more likely to be in the symptomatic group. A systematic review with meta-analysis investigated the prevalence of stress, anxiety, and depression in the general population during the first year of the pandemic across different continents (Salari et al., 2020). The prevalence of depression in 14 studies with a sample size of 44,531 was 33.7%. Additionally, Asia had the highest symptom rate at 35.3% (Salari et al., 2020).

A longitudinal study in Germany assessed changes in mental health symptoms, including depression, before and during the first and second waves of COVID-19. In the descriptive course, clinically relevant depression symptoms were more often reported in the first wave (13.9%), slightly decreasing in the second wave (12.1%), but did not reach pre-pandemic levels (9.7%). Depressive symptoms increased mainly in the younger individuals aged18 and 29 years (Hettich et al., 2022). Evidence has showed to other variables as relevant to understanding vulnerability to depressive symptomatology in the pandemic context, such as sex/gender (Qian et al., 2024), skin color/ethnicity (Maciel et al., 2023), and level of education (Zhu et al., 2023). Geographic region (Deng et al., 2021; Layton et al., 2021) and pandemic year (Grineski et al., 2024; Shen et al., 2024) are variables that have also been given attention and seem to predict the occurrence of depression.

Another study aimed to document the prevalence of depression in the adult population of the United States in the first year and 5 months of the pandemic based on data reported in peer-reviewed literature (Ettman et al., 2023). The overall mean prevalence reported over the first 17 months of the outbreak was 36.0% for mild depression, 26.0% for moderate depression, and 12.9% for severe depression. The researchers pointed out that the Patient Health Questionnaire-9 (PHQ-9) was the most used instrument to measure depressive symptoms and severity levels (*n* = 36; 76%; Ettman et al., 2023). These findings underscore the need to continue mapping the presence of depression in the population using context-adjusted measures so that with appropriate psychological strategies, techniques, and interventions, the mental wellbeing of the population can be maintained and improved.

### Measures in health psychology: what to pay attention to?

The post-pandemic period challenges research to better understand the events during the phenomenon and rely on valid and reliable measures that accurately represent the dynamics of what is being studied. Thus, evaluating the psychometric and predictive qualities of a measure becomes fundamental to verify its behavior (or functioning) during the pandemic. This process not only allows us to understand any necessary changes due to the context, if applicable, but also to ensure that its indicators have evidence of validity and can be used safely. The change in context requires identification and modification of measures adjusted to the period given the unique externalities of the experienced adversities (Hernández et al., 2020). Some groups or individuals may have been more likely to present significant symptomatology, and certain symptoms may have been more characteristic (Ettman et al., 2023). The ability of psychological and/or psychiatric instruments to capture this variability with necessary and validated robustness is essential to understand the dimensions of the pandemic’s impact on psychological adjustment.

To estimate the validity evidence of a measure – in this case, the PHQ-9 (Kroenke et al., 2001), one of the most common instruments for measuring depressive symptoms globally (Costantini et al., 2021) – specific predictors for depression screening in the population can be sought with greater reliability. This estimation would facilitate the formulation of a more complete assessment of the indicators. There is also the possibility of using the data in a metric manner (PHQ-9 scores), categorical levels (diagnoses for screening), or even stratification of symptom severity in a population. All of these uses are subject to different interests and applications in basic community clinics, individual clinical settings, or systemic interventions. Investing and refining the measure and its indicators can be considered a pertinent contribution to the study of depression during the pandemic and in the following years.

The PHQ-9 is one of the most used depression scale worldwide, having been adapted into multiple languages, including Spanish, Japanese, Russian, Portuguese, among others (Lamela et al., 2020). Its factorial structure has been extensively evaluated in different countries such as Norway (Wisting et al., 2021), Chile (Aslan et al., 2020), Argentina (Urtasun et al., 2019), Germany (Reich et al., 2018), even in the context of the COVID-19 pandemic, including the United States (Shaff et al., 2024), Brazil (Nunes and Faro, 2021), China (Gao and Liu, 2024). Most validity studies have found support for a single-factor structure, reinforcing the greater plausibility for the measure’s unidimensional model. The evidence demonstrates good psychometric quality of the instrument, proving it a robust measure with potential for cross-country result comparisons (Odero et al., 2023; Rahman et al., 2022).

### Proposal of the present study

This study aims to investigate different aspects related to the instrument, measure, and indicators of depression in the Brazilian population during the COVID-19 pandemic years 2020, 2021, 2022, and 2023. The first aim seeks to validate the use of the PHQ-9 in the pandemic, a widely disseminated measure for measuring depressive symptoms. The PHQ-9 has been used in different countries during the pandemic [China (Choi et al., 2020), Israel (Palgi et al., 2020), France (Peretti-Watel et al., 2020), the United Kingdom (Kotabagi et al., 2020), Australia (Stocker et al., 2021), USA (Sekimitsu et al., 2022), Japan (Fukase et al., 2021), among others], demonstrating its breadth of application. In Brazil, we relied on the study performed by (Nunes and Faro, 2022), which only covered the 2020. In recent years, other studies involving Brazilian participants have been conducted, but they have primarily focused on specific demographics, such as undergraduate students (Rufino et al., 2024), or were limited to a single city (Lua et al., 2022). These studies did not address the broader population during the pandemic period. Since that data collection, the pandemic has gone through different phases which can be understood as differentiated periods within each year. These periods could have altered aspects of psychometric equivalence of the measure. As a result, the variation requires a new study with these new characteristics and more recent data, thus we performed a confirmatory factor analysis (CFA) and multi-group CFA of the PHQ-9 of the Brazilian population considering samples from four different time periods of the pandemic. This analysis will evaluate the potential effect of the pandemic year on the outcome along with a larger and more diversified geographic sample.

The second aim assesses the potential to standardize the PHQ-9 scores in the sample using novel data. This analysis will allow other studies to have parameters to weigh their findings and thus assist in clinical, policy, or governmental decision-making. Although it is not a randomized sample, the number of participants (over 10,000) and regional diversity (around 1,500 cities) helps the purpose of comparison not for diagnostic strata but for establishing general cut score based on an extensive sample.

The third aim seeks to conduct predictive testing of the PHQ-9 to enhance the findings of the study including information about the vulnerability of certain groups or individuals to depression in Brazil. These data are novel considering the proposed national design. The regression models (linear and logistic) seek to estimate vulnerability of certain groups dependent on specific conditions providing greater direction for current and future public health interventions.

## Materials and methods

### Design’s study

The study had a successive cross-sectional and a non-probabilistic design based on independent samples by convenience method. Inclusion criteria included adults aged 18 years or more, and agreement to participate through a consent form. The exclusion criterion was an incomplete questionnaire response. The data were collected in four moments, one per year, in different years and distinct samples: the first from June 2 to June 6, 2020 (Epidemiological Week [EW], 23); at that moment, the restriction measures included social isolation and quarantine for the general population and at-risk groups, curfews, suspension of events and classes, transportation restrictions, mandatory mask-wearing, and economic shutdown. The second one was from March 18 to April 5, 2021 (EW 11, 12, and 13). In 2021, in addition to the previously mentioned measures, beach and park closures, prohibition of religious gatherings, collective sports activities, and food takeout from bars and restaurants were in effect. At that time, schools remained open only for meal distribution and provision of materials to students in need. Furthermore, national holidays were brought forward to encourage social isolation. The third data-collection was from March 17 to March 24, 2022 (EW 11 and 12). In 2022, the mandatory use of masks ceased in several states, such as Rio de Janeiro, Rio Grande do Sul, the Federal District, among others. All the others restriction measures were finished. The fourth data-collection was from March 17 to March 31, 2023 (EW 11, 12, and 13). In 2023, the use of masks was already optional nationwide and other restrictions were not in effect at the time.

### Participants

10,069 adults comprised the full sample. Participants were from all 27 Brazilian states and approximately 1,500 cities. The majority of the total sample was collected in 2020 (47.6%; *n* = 4,793), followed by 2023 (22.2%; *n* = 2,238). 2021 and 2022 had roughly the same number of participants (15.1%; *n* = 1,522; *n* = 1,516, respectively). Females constituted the majority (88.2%; *n* = 8,880), as well as people who identified as White individuals (54.4%; *n* = 5,481), and those who had higher educational attainment (undergraduate and graduate, 81.6%; *n* = 8,218). The age group with the most participants was 18–24 years old (27.0%; *n* = 2,722), while the smallest group was those over 55 years old (13.3%; *n* = 1,342). The Northeast and Southeast regions were the most represented (39.2%; *n* = 3,951 and 38.6%; *n* = 3,891, respectively), with the North having the smallest proportion in the sample (3.1%; *n* = 311). Based on a screening cut score (> 10), almost two-thirds of the sample presented some level of significant depressive symptomatology (63.2%; *n* = 6,362) (Table 1).

**Table 1:** General sample profile, Brazil (2020–2023).

Variables	F% (*n* = 10,069)
Year	
2020	47.6 (4,793)
2021	15.1 (1,522)
2022	15.1 (1,516)
2023	22.2 (2,238)
Sex/gender	
Male	11.8 (1,189)
Female	88.2 (8,880)
Skin color/ethnicity	
White individuals	54.4 (5,481)
Black individuals	9.7 (975)
*Parda* (mixed race) individuals	35.9 (3,613)
Education level	
High school	18.4 (1,851)
Higher school	81.6 (8,218)
Region	
North	3.2 (311)
Northeast	39.2 (3,951)
Midwest	5.2 (528)
South	13.8 (1,388)
Southeast	38.6 (3,891)
Age group	
18–24	27.0 (2,722)
25–34	24.6 (2,473)
35–44	20.5 (2,066)
45–54	14.6 (1,466)
More than 55	13.3 (1,342)
Depressive symptomatology (screening diagnosis, score > 10)	
No	36.8 (3,707)
Yes	63.2 (6,362)

### Instruments

The PHQ-9 is a self-administered questionnaire used for screening the presence and severity of depressive symptoms over the last 2 weeks, based on the Diagnostic and Statistical Manual of Mental Disorders, 5th edition (DSM-V) criteria (Kroenke et al., 2001). The PHQ-9 consists of nine items, with responses using a four-point Likert scale (0 = never, 1 = several days, 2 = more than half the days, 3 = nearly every day). According to the original study, a score higher than 10 indicates the presence of depressive symptoms. Previous research has demonstrated good psychometric properties of the scale (pre-pandemic studies), even during the pandemic period (Kroenke et al., 2001; Nunes and Faro, 2022; Odero et al., 2023; Sun et al., 2020). The Cronbach’s alpha (*α*) and McDonald’s omega (*Ω*) found in the current study were 0.90, indicating excellent internal consistency.

The Generalized Anxiety Disorder Screener (GAD-7) is a brief self-report measure that assesses the frequency of anxiety signs and symptoms over the past two weeks (Spitzer et al., 2006). The instrument consists of seven items rated on a four-point scale ranging from 0 (not at all) to 3 (nearly every day). The total score is obtained by summing the item responses, ranging from 0 to 21 points, with a score of 10 or higher considered a probable indicator of Generalized Anxiety Disorder (GAD). In its original study, the GAD-7 demonstrated excellent internal consistency (Cronbach’s *α* = 0.92). In this study, reliability was 0.90. A sociodemographic questionnaire was administered to collect data regarding the sample characteristics. Participants provided information about their age (in years, but later stratified into age groups), the year of the survey (2020, 2021, 2022 or 2023), sex/gender (male, female or other option) skin color/ethnicity (White, Black, *Parda* [mixed race], Yellow or Indigenous individuals, or other options), region of the country in which they reside (North, Northeast, Midwest, South, and Southeast), and education level (up to High school and Higher school).

### Procedures and ethical considerations

This study was authorized by the Brazilian Research Ethics Committee with Human Subjects (National Council of Ethics and Research [CONEP], n° 3.955.180). For 4 years, data collection was done by approaching participants through invitations on digital platforms such as WhatsApp and Instagram. The questionnaire was created using the Survey Monkey platform and made available to individuals who accepted the invitations. It was not possible to estimate how many individuals did not agree to participate due to the method of randomly boosting the invitations in the social media platform. A consent form was presented on the first page of the questionnaire. Participants were granted access to the research questionnaires only after consent.

### Data analysis

For the CFA and measurement invariance (MI), the Robust Diagonally Weighted Least Squares (RDWLS) estimation method was adopted. These analyses were performed using JASP Software (Version 0.18.0). The fit indices included the Comparative Fit Index (CFI; expected >0.95), Tucker-Lewis Index (TLI; expected >0.95), Root Mean Square Error of Approximation (RMSEA; expected ≤0.050, CI [95%] between 0 and 0.08; *p*-close >0.05) and Standardized Root Mean Residual (SRMR; expected ≤0.080). The testing for MI was executed at four levels: configural, metric, scalar, and strict (Cheung and Rensvold, 2002). The criteria of ΔCFI <0.010 and ΔRMSEA <0.015 were set for sequential models to assess invariance (Chen, 2007). Reliability was analyzed using Cronbach’s alpha (expected >0.60) and McDonald’s omega (expected >0.70) (Hair et al., 2014). The T Score (T = 50 + 10Z) of the PHQ-9 scores was also calculated.

Hierarchical linear regression (LinR) and binomial logistic regression (LogR) were executed through JAMOVI software (The jamovi project, 2022). The PHQ-9 score was the dependent variable in two types: score (LinR) and screening diagnosis (LogR). Sociodemographic and pandemic-related variables were added as independent variables in progressive blocks, as follows: pandemic year (2020, 2021, 2022 and 2023), sex/gender (male and female), skin color/ethnicity (White individuals, Black individuals and *Parda* [mixed race] individuals), education level (high school and higher school), Region (North, Northeast, Midwest, South and Southeast), and age group (18–24, 25–34, 35–44, 45–54 and more than 55 years old). Yellow, Indigenous individuals or other options for skin color/ethnicity, and other options for sex/gender participants were excluded of the sample due to the low quantity (<0.1%). Fit indicators included *R*^2^, adjusted *R*^2^, delta *R*^2^ (Nagelkerke’s *R*^2^ for LogR) to identify individual contributions of the independent variables to the models (LinR and LogR). The Durbin-Watson test and tests for evaluating multicollinearity (VIF and Tolerance) were analyzed (LinR). A Pearson correlation test between PHQ-9 (skewness = 0.046 and Kurtosis = −1.090) and GAD-7 (skewness = 0.046 and Kurtosis = −0.081) was performed to examine evidences of convergent validity. The significance level for the analyses in this study was set at *p* < 0.05.

## Results

### Confirmatory factor analysis

The CFA general model showed a unidimensional structure of the PHQ-9. The fit indices were considered satisfactory [TLI = 0.99; CFI = 0.99; RMSEA = 0.05 (CI 95% = 0.047–0.054); SRMR = 0.003] and the total explained variance (*R*^2^) by item varied from 0.271 (item 9) to 0.708 (item 2), with an average of 0.522. All items loaded above 0.500, ranging from 0.520 (item 9) to 0.841 (item 2) with a mean loading as high as 0.717 (Table 2). Models fit at all four different levels of invariance (configural, metric, scalar, and strict levels; ∆CFI and ∆RMSE criteria) in relation to year, gender, educational level, geographic region, skin color, and age group comparisons (Table 3).

**Table 2:** Confirmatory factor analysis (factor loadings) and descriptive data of the Patient Health Questionnaire-9 items (PHQ-9), Brazil (2020–2023).

Items	λ	%	M (SD)	Skew	Kurt
1.Little interest or pleasure in doing things.	0.78	84.0	1.6 (1.07)	−0.06	−1.30
2.Feeling down, depressed, or hopeless.	0.84	85.2	1.7 (1.09)	−0.05	−1.37
3.Trouble falling or staying asleep or sleeping too much.	0.71	83.5	1.8 (1.15)	−0.25	−1.43
4.Feeling tired or having little energy.	0.79	91.3	1.9 (1.05)	−0.32	−1.32
5.Poor appetite or overeating.	0.69	79.7	1.7 (1.17)	−0.16	−1.48
6.Feeling bad about yourself or that you are a failure or have let yourself or your family down.	0.75	72.1	1.5 (1.21)	0.05	−1.57
7.Trouble concentrating on things, such as reading the newspaper or watching.	0.72	76.6	1.5 (1.14)	0.11	−1.40
8.Moving or speaking so slowly that other people could have noticed. Or so fidgety or restless that you have been moving a lot more than usual.	0.63	52.4	1.0 (1.01)	0.77	−0.78
9.Thoughts that you would be better off dead, or thoughts of hurting yourself in some way.	0.52	31.0	0.5 (0.95)	1.63	1.37
**Total score**	–	63.2*	13.2 (7.51)	0.04	−1.09

λ, factor loadings; M, mean; SD, standard deviation; %, percentage of subjects indicating response options 1, 2, or 3 (presence of symptom); Skew, skewness; Kurt, kurtosis. * Percentage of individuals with scores above the cut score of 10.

**Table 3:** Measurement invariance analysis of the unidimensional model of PHQ-9, Brazil (2020–2023).

Parameters	Sex/gender	Education level	Year	Region	Skin color/ethnicity	Age group
**χ** ^**2**^ **(gl)**	–	–	–	–	–	–
*Configural*	718.013 (54)	719.115 (54)	760.155 (108)	762.046 (135)	743.231 (108)	715.437 (135)
*Metric*	785.515 (62)	791.320 (62)	907.144 (132)	942.736 (167)	790.140 (132)	1192.952 (167)
*Scalar*	836.943 (70)	811.978 (70)	1089.156 (156)	973.208 (199)	808.642 (156)	1361.594 (199)
*Strict*	918.841 (79)	857.317 (79)	1185.848 (183)	1116.103 (235)	845.181 (183)	1735.774 (235)
**CFI**	–	–	–	–	–	–
*Configural*	0.994	0.994	0.994	0.995	0.995	0.994
*Metric*	0.994	0.994	0.993	0.993	0.994	0.990
*Scalar*	0.993	0.994	0.992	0.993	0.994	0.989
*Strict*	0.993	0.993	0.992	0.992	0.994	0.986
**∆CFI**	–	–	–	–	–	–
*Configural*	–	–	–	–	–	–
*Metric*	0	0	0	0.002	0.001	0.004
*Scalar*	0.001	0	0	0	0	0.001
*Strict*	0	0.001	0	0.001	0	0.003
**RMSEA**	–	–	–	–	–	–
*Configural*	0.049	0.049	0.049	0.048	0.048	0.048
*Metric*	0.048	0.048	0.048	0.048	0.045	0.055
*Scalar*	0.047	0.046	0.049	0.044	0.041	0.054
*Strict*	0.046	0.044	0.047	0.043	0.038	0.056
**∆RMSEA**	–	–	–	–	–	–
*Configural*	–	–	–	–	–	–
*Metric*	0.001	0.001	0.001	0	0.003	−0.007
*Scalar*	0.001	0.002	−0.001	0.004	0.004	0.001
*Strict*	0.001	0.002	0.002	0.001	0.003	−0.002

Sex/Gender: Male or Female; Education: High school or higher education; Year (2020, 2021, 2022, and 2023), Skin color/Ethnicity (White, Black and *Parda* [mixed race] individuals), Region (North, Northeast, Midwest, South, and Southeast), Age group (18–24, 25–34, 35–44, 45–54 and more than 55 years old). χ^2^, Chi-square; df, degrees of freedom; CFI = Comparative Fit Index; RMSEA = Root Mean Square Error of Approximation; ∆ = Delta. ∆CFI (expected ≤ 0.01); ∆RMSEA (expected ≤ 0.015).

**Figure 1:**
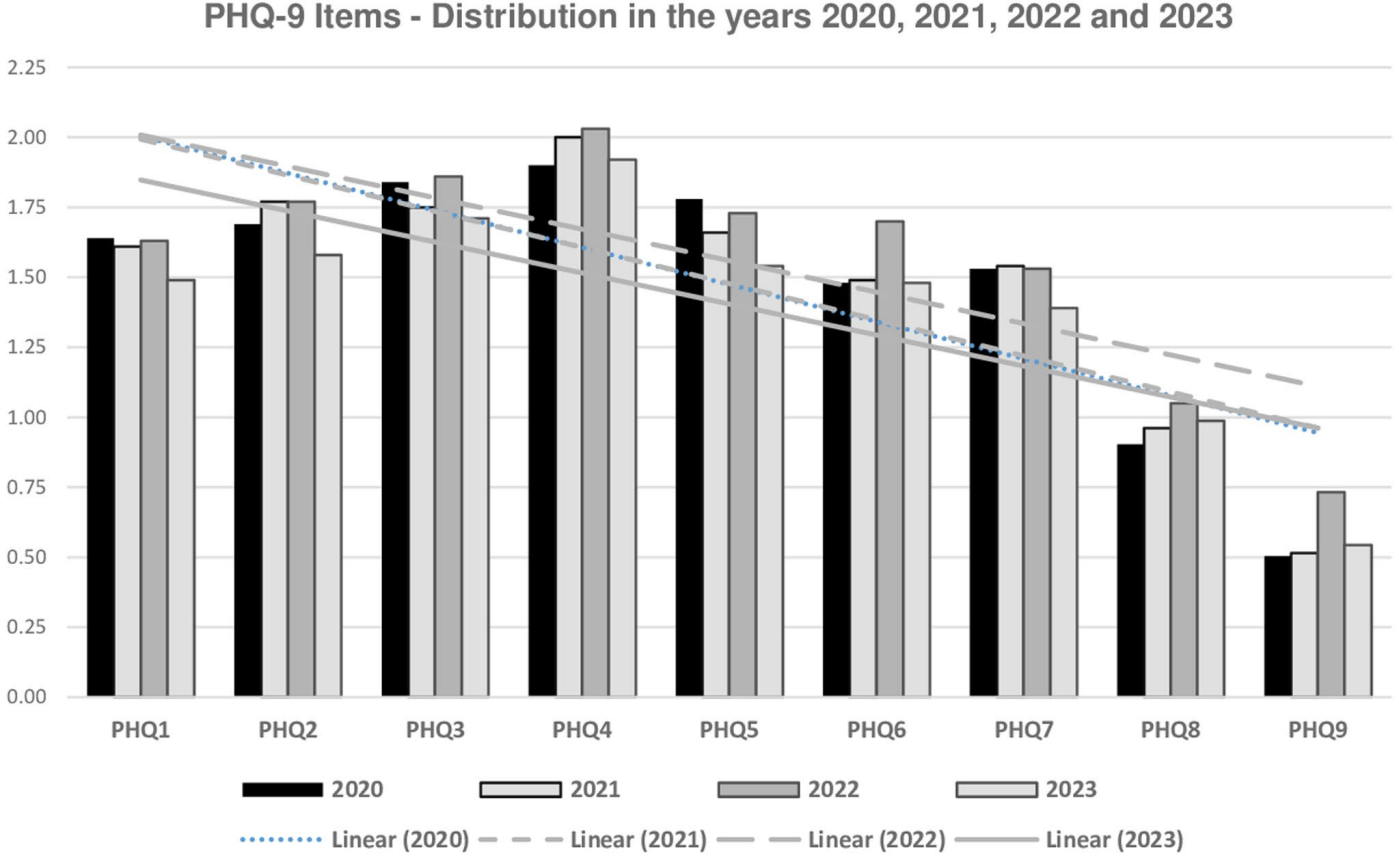
Distribution of PHQ-9 items, Brazil (2020–2023, *n* = 10,069). PHQ1, interest or pleasure; PHQ2, disinterest; PHQ3, sleep disturbances; PHQ4, fatigue; PHQ5, appetite changes; PHQ6, negative self-esteem; PHQ7, concentration difficulties; PHQ8, psychomotor changes; PHQ9, suicidal ideation.

The descriptive data on the PHQ-9 items are displayed in Figure 1. Item 4 (Tiredness or lack of energy) showed the highest mean, followed by items 3 (had difficulty falling asleep or staying asleep without interruptions or slept too much), 2 (felt down, depressed, or hopeless), and 5 (had a poor or excessive appetite). The lowest were items 9 (thoughts that you would be better off dead, or of hurting yourself in some way) and 8 (Moved or spoke so slowly that other people could have noticed). The most frequent symptoms were item 4 (91.3%), item 2 (85.2%), and item 1 (had little interest or pleasure in doing things). The least common items were 9 (31.0%) and 8 (52.4%). The distribution of the item means from 2020 to 2023 is represented in Figure 1. A positive correlation between PHQ-9 and GAD-7 scores was found (Pearson = 0.756; *p* < 0.001), indicating the higher the depression score, the higher the anxiety score.

### Normative score for PHQ-9 (Brazil, 2020–2023)

Based on the CFA and CFAMG findings, Table 4 was created to propose parameters for estimating various scores based on the distribution in the total sample. Table 4 presents the T score for making comparisons on a standardized scale, the frequency of each score in the current population, and the cumulative frequency. Both the T Score and cumulative frequency provide support for hypothetical cut score indicating the severity of depressive symptoms in the general population. These suggestions are based on the social distribution of the PHQ-9 and can be a possible method for screening differences between samples or scenarios (e.g., different time-points after the COVID-19 pandemic). Therefore, the strata can be categorized as follows: very low (score 0 to 6, T = 40; F%cum ≈ 20%), low (score 7 to 13, T = 50; F%cum ≈ 50%), moderate (score 14 to 19, T = 58; F%cum ≈ 75%), high (score 20 to 23, T = 63; F%cum ≈ 90%), and very high (score > 24, T > 63; F%cum > 93.1%).

**Table 4:** Standardization of PHQ-9 scores during the COVID-19 pandemic, Brazil (cross-sectional data from 2020 to 2023, *n* = 10,069).

Score	Score *T*	Total *F*%	Cumulative *F*%
0	32	2.9	2.9
1	34	2.2	5.1
2	35	2.7	7.8
3	36	3.2	11.0
4	38	3.7	14.7
5	39	4.0	18.7
6	40	4.2	22.9
7	42	4.6	27.5
8	43	4.6	32.1
9	44	3.9	35.9
10	46	4.2	40.1
11	47	4.0	44.1
12	48	3.8	47.9
13	50	3.5	51.4
14	51	3.9	55.3
15	52	3.8	59.0
16	54	3.8	62.9
17	55	4.3	67.1
18	56	4.3	71.4
19	58	3.6	75.0
20	59	3.8	78.7
21	60	3.7	82.4
22	62	3.7	86.1
23	63	3.4	89.5
24	64	3.5	93.1
25	66	2.4	95.5
26	67	1.9	97.4
27	68	2.6	100.0

Up to 6 points: very low (T = 40; cumulative F% = 2.9–22.9%), between 7 and 13 points: low (T = 50; cumulative F% = > 22.9–51.4%), between 14 and 19 points: moderate (T = 58; cumulative F% = > 51.4–75.0%), between 20 and 23 points: high (T = 63; cumulative F% > 75.0–89.5%), above 24 points: very high (T > 63 up to 68; cumulative F% > 93.1–100.0%).

### Multiple linear regression analysis

In the final adjusted linear regression model, all previous blocks showed statistical significance. In comparison to 2020, 2021 and 2022 exhibited a higher probability of being related to an increase in depressive symptomatology (*β* = 0.103 and 0.215, respectively; *p* < 0.001). There was no difference between 2020 and 2023 (*p* > 0.05). Females had greater depressive symptomatology compared to males (β = 0.345; *p* < 0.001). Participants identified as *Pardos* indicated more depressive symptoms than White individuals (β = 0.106; *p* < 0.001), but no difference was found when contrasting Black and White respondents (*p* > 0.05). Participants who self-reported having up to a high school education were more prone to show higher scores of depression symptoms (β = 0.132; *p* < 0.001). People living in the Northeast showed lower scores than those in the South (β = −0.342; *p* < 0.001); no other comparison by region was statistically significant (*p* > 0.05). Age indicated statistical significance overall with the younger age of the participant corresponding to a higher their score in depressive symptomatology (from β = 0.310 for 45–54; to 0.900 for 18–24 years old; age more than 55 as reference; *p* < 0.001) (Table 5).

**Table 5:** Linear regression to the symptomatology of depression, PHQ-9, Brazil (cross-sectional data from 2020 to 2023, *n* = 10,069).

Variables (Step 6)	*R* ^2^_adj_	Δ*R*^2^	B	SE	*β* (CI 95%)	*p*-value
Year	0.002	–	–			
2021–2020			0.776	0.219	0.103 (0.046; 0.161)	<0.001
2022–2020			1.615	0.216	0.215 (0.158; 0.027)	<0.001
2023–2020			0.293	0.199	0.390 (−0.012; 0.090)	0.140
Sex/gender	0.015	0.013				
Female–Male			2.589	0.220	0.345 (0.287; 0.402)	<0.001
Skin color/ethnicity	0.016	0.001				
*Parda*-White individuals			0.795	0.252	0.106 (0.040; 0.172)	0.002
Black-White individuals			0.109	0.161	0.015 (−0.028; 0.057)	0.499
Education level	0.023	0.007				
High school-Higher school			0.991	0.192	0.132 (0.082; 0.182)	<0.001
Region	0.040	0.017				
North–South			0.224	0.507	−0.047 (−0.165; 0.070)	0.426
Northeast-South			−1.991	0.330	−0.342 (−0.405; −0.280)	<0.001
Southeast-South			0.604	0.330	0.002 (−0.055; 0.061)	0.925
Midwest-South			0.584	0.366	0.077 (−0.173; 0.017)	0.111
Age group	0.112	0.072				
18–24 – +55			6.761	0.252	0.900 (0.834; 0.966)	<0.001
25–34 – +55			4.857	0.249	0.647 (0.581; 0.711)	<0.001
35–44 – +55			3.988	0.251	0.531 (0.466; 0.567)	<0.001
45–54 – +55			2.302	0.268	0.310 (0.236; 0.377)	<0.001

Durbin-Watson = 1.96; VIF: lowest 1.01 – highest 1.05; tolerance = lowest 0.965 – highest 0.991; F_(last step)_ = 203.07 (df^1^ = 4; df^2^ = 10,053). *p* < 0.001. Omnibus test ANOVA: year, F = 19.64, *p* < 0.001; sex/gender, F = 137.95, *p* < 0.001; skin color/ethnicity, F = 5.00, *p* = 0.007; educational level, F = 26.70, *p* < 0.001; Region, F = 61.17, *p* < 0.001; age group, F = 203.07, *p* < 0.001. Residuals were normally distributed.

Considering the total explained variance of the final model (*R*^2^ converted to a percentage; 11.2%), the highest individual explained variance was related to age group, accounting for 7.2%. Skin color/ethnicity was the least powerful predictor (0.1%). In the middle were region (1.7%), education level (0.7%), sex/gender (1.3%), and year (0.2%) (Table 5).

### Adjusted binomial logistic regression analysis

The adjusted logistic regression was based on the binomial outcome of the PHQ-9 (score > 10 for the presence of significant depressive symptomatology at the screening level). The sixth block showed that all variables remained statistically significant (*p* < 0.001), with at least one categorical group showing statistical significance when contrasting has or not depression symptoms. The explained variance ranged from 0.012 (1.2%) in the first block to 0.127 (12.7%) in the last one. Within the variables, there were some exceptions: Black and White individuals (*p* = 0.599), and the regions North, Southeast, and Midwest versus South (*p* = 0.180, 0.580, and 0.138, respectively) did not show statistical significance (*p* > 0.05). All the other comparisons had a *p*-value less than 0.05. Table 6 displays the results, adjusted Odds Ratios (OR_adj_), and other indices of the logistic regression.

**Table 6:** Logistic regression to the symptomatology of depression, PHQ-9, Brazil (cross-sectional data from 2020 to 2023, *n* = 10,069).

Variables (Block 6)	*R*^2^N	Δ*R*^2^N	*p*-value	OR_adj_ (CI 95%)
Year	0.012	–		
2021–2020			< 0.001	1.275 (1.115; 1.145)
2022–2020			< 0.001	1.409 (1.234; 1.610)
2023–2020			< 0.001	0.796 (0.708; 0.896)
Sex/Gender	0.024	0.012		
Female–Male			< 0.001	1.900 (1.670; 2.161)
Skin Color/Ethnicity	0.026	0.002		
*Parda*-White individuals			0.005	1.252 (1.071; 1.462)
Black-White individuals			0.599	1.026 (0.932; 1.131)
Education level	0.032	0.024		
High school-Higher school			< 0.001	1.272 (1.130; 1.432)
Region	0.053	0.021		
North–South			0.180	0.823 (0.619; 1.094)
Northeast-South			< 0.001	0.470 (0.406; 0.544)
Southeast-South			0.580	0.962 (0.837; 1.105)
Midwest-South			0.138	0.842 (0.671; 1.057)
Age group	0.127	0.074		
18–24 – +55			< 0.001	5.666 (4.859; 6.607)
25–34 – +55			< 0.001	3.384 (2.922; 3.198)
35–44 – +55			< 0.001	2.654 (2.293; 3.071)
45–54 – +55			< 0.001	1.716 (1.472; 2.000)

OR_adj_, Adjusted Odds Ratio values; *R*^2^N, Nagelkerke’s *R*^2^ for the block; Δ*R*^2^N, Delta Nagelkerke’s *R*^2^ for the specific variable added to the last block. VIF: highest 1.05 – lowest 1.01; tolerance: highest 0.988 – lowest 0.953. Accuracy of the predictive model: 67.1% (0.671).

2020 was the reference group, and all three other years presented significant differences as follows: 2021 and 2023 were more prone to have more cases of depressive symptomatology (OR_adj_ = 1.275 [27.5%] and 1.409 [40.9%], respectively; *p* < 0.001). When comparing 2023 to 2020, there was a decrease in the prevalence of depressive symptomatology of around 20% (OR_adj_ = 0.796; *p* = 0.140). Females were had almost two times greater prevalence of depressive symptomatology than males (OR_adj_ = 1.900 [90.0%]; *p* < 0.001). *Pardos* individuals showed more likelihood of those with symptoms of depression (OR_adj_ = 1.252 [25.2%]; *p* = 0.002) compared to White individuals. People who self-reported having only a high school education had a 27.2% (OR_adj_ = 1.272; *p* < 0.001) higher chance of being part of the depression group. The only significant regional difference was between Northeast and South; the former had almost half the chance of the latter (OR_adj_ = 0.470; *p* < 0.001). Age group had a progressive increase of top-down chances (from OR_adj_ = 1.716 to 5.666; all contrasts at *p* < 0.001): the younger the strata, the higher the chances of being part of the group with depressive symptomatology (Table 6).

The Delta *R*^2^ of Nagelkerke indices arranged in descending order of importance with the percentage of individual explained variance by variable demonstrated age group as the most powerful (7.4%), followed by education level (2.4%), region (2.1%), sex/gender and Year (both 1.2%), and skin color/ethnicity (0.2%).

## Discussion

This study aimed to investigate different aspects related to the instrument, measure, and indicators of depression in the Brazilian population during the COVID-19 pandemic years of 2020, 2021, 2022, and 2023. We also assessed validity of the PHQ-9 by analyzing the internal structure of the measure (CFA and invariance analysis) and characterized the sample based on sociodemographic variables. This investigation sought to standardize PHQ-9 to establish cut score based on the obtained sample. Parameters of social distribution were then presented to estimate greater vulnerability to screening diagnoses.

Considering parameters suggested by Kroenke et al. (2001), over 60% of the sample had a positive screening diagnosis for depression, meaning that six out of every ten individuals exhibited depressive symptoms. The presence of depressive symptoms tends to be associated with functional impairment, reduced quality of life, and a higher risk of mortality (Ribeiro et al., 2020), in addition to an increased risk of suicide (Richie et al., 2021). This trend was found in studies on depression in Brazilian samples (Barros et al., 2020; Pereira-Ávila et al., 2021). Similar results were observed in studies from other countries as well (Choi et al., 2020; Clarke-Deelder et al., 2022; Fanaj and Mustafa, 2021). In comparison, a lower occurrence of depression was noted in research conducted before the pandemic (Martins-Monteverde et al., 2019; Nunes et al., 2022). Therefore, detecting that 60% of the participants in this study exhibited potential vulnerability to depression underscores indicating the need to monitor mental health outcomes in the post-pandemic period and to develop effective interventions to address current symptoms (Cataldo et al., 2023).

Similar to previous studies, we confirmed that the PHQ-9 exhibits a unidimensional structure (Fonseca-Pedrero et al., 2023; Mwangi et al., 2020; Pranckeviciene et al., 2022). This finding corroborates a previous investigation (Nunes and Faro, 2022) of the dynamics of the phenomenon throughout the pandemic. The aim was to expand the repertoire of statistical analyses to present greater robustness of the evidence for the validity of the instrument by standardizing the PHQ-9 scores and including other variables of interest in multi-group CFA (year, geographic region, skin color, and age). This investigation confirms the single-factor structure of the measure but includes a larger sample and temporal scope (different years of the pandemic). It is important to note that we did not aim to perform comparisons between CFA models (alternative factor structures) in the current study. We adopted the unidimensional structure, which has been used abroad (i.e., Bianchi et al., 2022; Boothroyd et al., 2019), including recent studies during the COVID-19 pandemic (i.e., Choi et al., 2020; Fukase et al., 2021; Kotabagi et al., 2020; Magalhaes et al., 2021; Palgi et al., 2020; Peretti-Watel et al., 2020; Sekimitsu et al., 2022; Stocker et al., 2021). However, it’s worth noting that there is no consensus on the ideal factor structure (e.g., two-factor structure, Lamela et al., 2020; four-factor structure, Tseng et al., 2024). We hope this decision can support direct comparisons between data from Brazil and other countries regarding the pandemic.

One of the innovations of this study was the standardization of PHQ-9 to establish cut scores to assess the severity levels of depressive symptoms. The suggested cut scores were established based on the social distribution of the PHQ-9; thus, it is not an analysis of criterion validity evidence. The establishment of cut scores and determination of the T score allow for the parameterization of the distribution of scores of this measure in other contexts as well as enabling the comparison of different research scenarios. For example, it would be possible to track the evolution of symptomatology over time at different points after the pandemic. The cut scores were established as follows: very low (score 0 to 6), low (score 7 to 13), moderate (score 14 to 19), high (score 20 to 23), and very high (score > 24). Consequently, it would be possible to map severity levels and identify associated vulnerabilities in different contexts. This approach enables comparisons with other regions or even other countries considering the diversity of coping responses adopted throughout the COVID-19 crisis.

An overview on the findings shows that the sex, skin color, age, education level, and year were predictors of depressive symptoms. Females, those who self-identified as Pardo, younger individuals, and those with education up to high school level had higher scores. The years 2021 and 2022 were more likely to be associated with an increase in depressive symptomatology.

Individual and social sex differences in depression occurrence are among the most robust and consistent findings in health Psychology research and represent a significant concern for healthcare professionals and researchers (Eid et al., 2019). As with other studies, we found no significant differences during the pandemic (Bucciarelli et al., 2022; Cha et al., 2022; Jeong et al., 2023). The findings of this investigation add valuable information and substantiate sex differences concerning concerns during COVID-19, prioritizing assessing females for depressive symptomatology (Ausín et al., 2021). Other research conducted during the pandemic indicated that women are among the most psychologically affected groups, demonstrating that the psychological impact during the COVID-19 outbreak was greater in this group, although men were also affected (Flentje et al., 2020). Since the early studies on the psychological effects of the pandemic, women have been found to be more affected with greater negative alterations in cognition or mood than men (Rania and Coppola, 2021). One possible explanation could be that women might be more susceptible to depression risk factors and more susceptible to the negative effects of social loneliness (Liu et al., 2020). Women might also perceive the pandemic period as more threatening and feel they lack sufficient resources to cope with the situation (Flentje et al., 2020). Regarding social factors, the pandemic context has reinforced structural gender inequality and differences in the roles played by men and women, which is a possible explanatory factor in understanding women’s greater vulnerability to depressive symptoms (Kolakowsky-Hayner et al., 2021). It is worth noting that men often omit their symptoms of depression due to the fear that it may affect their masculinity, resulting in a potential underestimation of the prevalence and underdiagnosis of depression in this group (Shi et al., 2021). Therefore, gender disparities reiterate a crucial public health concern, emphasizing the importance of prioritizing psychological support measures for more vulnerable groups.

Younger individuals were also more susceptible to depression during the pandemic, corroborating other findings in the literature (Robb et al., 2020; Varma et al., 2021). This finding aligns with previous studies on COVID-19-related effects on mental health across different age groups, where younger individuals seem to be at higher risk of increased depression compared to older adults (Haigh et al., 2018; Weinberger et al., 2018). Evidence indicated that depressive symptoms tend to be higher in younger adults, decreasing through mid-adulthood, and increasing again in older age (Hargrove et al., 2020). The trajectory of the disease became more prominent throughout life due to the severity of symptoms among young adults. In other words, the presence of higher levels of depression during this life stage was a contributing factor to the course of the disorder in other stages of development. Some explanations suggest that older adults are more resilient to adversities, worry less, and possess superior emotional regulation and coping strategies (Liu et al., 2022).

The years 2021 and 2022 had a higher likelihood of presenting elevated depression scores, consistent with other investigations (Hajek et al., 2022; Rudenstine et al., 2022). Despite advancements in dealing with COVID-19 and the increasing, albeit uneven, availability of vaccines, 2021 was the deadliest year of the pandemic worldwide. Approximately 3.9 million deaths occurred in 2021, accounting for nearly half of all COVID-19 deaths from 2020 to 2022. Brazil followed this global trend, with the year 2021 being the deadliest period of the pandemic in the country, which could help to explain the increase in mental health outcomes, including levels of depression. The years 2021 and 2023 were more likely to have more cases of depression. These results suggest a delayed effect of the psychological impacts of the pandemic. Given that it is a chronic stressor event, it is expected that different adaptive responses may be established later, considering the adaptive wear and tear experienced in the preceding pandemic years (Park et al., 2021).

Lower levels of education have been associated with depression occurrence within (Peng et al., 2020; Song et al., 2021) and before (Nunes et al., 2022; Lotfaliany et al., 2018) the pandemic period. Our findings corroborate these studies as participants with up to a high school education were more vulnerable to depressive symptomatology. One possible explanation is that individuals with higher education may perceive and cope with the pandemic’s impact more rationally, favoring adaptation compared to those with lower levels of education (Song et al., 2021). Furthermore, higher education level is likely to provide individuals with social and economic resources to cope with the pandemic whereas lower education levels eventually limit access to social and economic resources, which puts those at a higher risk (Abrams and Szefler, 2020). Individuals identifying as *Pardo* (mixed race) had greater depressive symptomatology. Another study conducted in Brazil found similar results where individuals identifying as *Pardo* had a 23% higher prevalence of symptoms than those who identified as White individuals (Santos et al., 2021). Literature suggests that non-White populations are more likely to suffer the impacts of the pandemic due to historical neglect resulting in greater social and psychological vulnerability (Dickinson et al., 2021). Our results indicate the same.

Individuals from the Northeast region were more vulnerable to depression compared to those from the South region. Although there is no direct explanation in the literature for this association (region x diagnosis), this finding appears to align with the relationship between depression and social and economic inequalities. A study on the effects of COVID-19 in the Northeast region suggested that despite the implementation of public health measures, the crisis exacerbated existing inequalities in the region leading to not only a significant number of cases and deaths, but also an increase in poverty and the growth of racial and ethnic disparities (Kerr et al., 2020). Thus, such scenario is a condition that influences the occurrence of depression (Proto and Quintana-Domeque, 2021).

Clinical implications of this investigation can be significant for public mental health services in Brazil and other similar countries, such as Low- and Middle-Income Countries (LMICs), particularly in the context of ongoing and future pandemic crises. Our findings confirm the suitability of the PHQ-9 as a reliable instrument, and its standardization for assessing depressive symptoms across diverse populations and time points during the COVID-19 pandemic revealed a relevant portrait. By identifying specific sociodemographic predictors, the study also helps to provide critical insights focused on the groups that showed higher vulnerability to significant depression symptoms. For instance, this information can guide the development of targeted interventions, such as mental health campaigns, policies, and support programs aimed at addressing these vulnerabilities (Arias de la Torre et al., 2023; Lund, 2023; Mughal et al., 2023; Terman et al., 2023). Furthermore, the establishment of normative cut scores for depressive symptom severity enables more accurate monitoring and comparison of mental health trends over time, facilitating early identification and treatment (GBD 2019 Mental Disorders Collaborators, 2022; Lattie et al., 2022; Shevlin et al., 2022; Witteveen et al., 2023). These insights highlight the need for integrating robust mental health strategies into broader public health responses to mitigate the psychological impact of health crises in the face of future global health challenges.

A question to consider is what factors may contribute to the maintenance of depressive symptoms within the context of the COVID-19 pandemic. Evidence from studies highlights the importance of dysfunctional coping strategies, such as perseveration, which includes repetitive behaviors like excessive checking, constant worrying, and monitoring driven by fear or perceived threats related to COVID-19 (Mansueto et al., 2022). Additionally, psychological inflexibility, which impairs individuals’ ability to adapt to challenging circumstances and regulate their emotional responses, has been linked to increased severity of depressive symptoms (Mansueto et al., 2024). Therefore, such behavioral, emotional, and cognitive patterns underscore how maladaptive responses to pandemic-related stressors can sustain or exacerbate depressive symptoms, emphasizing the need for targeted interventions.

Limitations of this study include a non-probabilistic and convenience-based sample design despite extensive geographical coverage. As such, it is not possible to generalize to the Brazilian population especially with the majority of sample being predominantly adult females with a higher level of education (completed or not) from the Northeast and Southeast regions of the country. The findings of this study provide no insights to other populations that are not adults, such as children, adolescents, or the elderly. Other limitations concern the fact that respondents were invited to participate in the research through social networks, which probably implies a reduced number of individuals over 60 years old in the sample, as the majority of the elderly population does not use social media platforms as younger generations. It should be also noted that the variable sex/gender was lastly used with only two groups (men and women). This may restrict the generalization of the results under a broader gender diversity, as the experiences and perspectives of individuals with non-binary, transgender, or other groups were not evaluated.

Future research should replicate this study with a more diversified and representative sample of the Brazilian population. Furthermore, another point require attention; for example, prior health issues such as mental health problems or pharmacological treatment, and other life events that could influence the presence of depressive symptoms were not explored. It is recommended that future studies address these gaps to map other aspects that assist in better understanding the occurrence of depression.

Finally, the use of PHQ-9 to measure depressive symptomatology in Brazil is recommended as it is a short instrument, easy to understand, apply, and score. The prevalence of depressive symptomatology was considered high overall and consistent with the varying impact of pandemic on the psychological adjustment in the general population by age, sex, and education level. Based on the assessment of PHQ-9 normative data, we believe is possible to monitor its social distribution and dynamic changes in the coming years. This study adds novel findings regarding the predictive ability to monitor these changes over time and prevent it from fading into partial, perhaps traumatic, resolution without new adaptive skills developed for future crises. It also has present implication considering the COVID-19 pandemic is still ongoing in 2023, albeit attenuated. Epidemiological projections predict new pandemics will come even if not on the same scale. Given the magnitude of these findings from a mental health perspective, future studies should ask: *(a) What have we learned from dealing with the COVID-19 pandemic? and (b) Are we prepared for the next one or at least beginning to prepare?*

## Data Availability

The raw data supporting the conclusions of this article will be made available by the authors, without undue reservation.
